# Quantification of stimulus-evoked tactile allodynia in free moving mice by the chainmail sensitivity test

**DOI:** 10.3389/fphar.2024.1352464

**Published:** 2024-02-23

**Authors:** Yildirim Ozdemir, Kazuo Nakamoto, Bruno Boivin, Daniel Bullock, Nick A. Andrews, Rafael González-Cano, Michael Costigan

**Affiliations:** ^1^ The Department of Anesthesiology, Critical Care and Pain Medicine, Boston Children’s Hospital, Harvard Medical School, Boston, MA, United States; ^2^ Department of Clinical Pharmacy, School of Pharmaceutical Sciences, Kobe Gakuin University, Kobe, Japan; ^3^ In Vivo Scientific Services, The Salk Institute for Biological Studies, La Jolla, CA, United States; ^4^ Department of Pharmacology, Faculty of Medicine and Biomedical Research Center (Neurosciences Institute), Biosanitary Research Institute ibs.GRANADA, University of Granada, Granada, Spain

**Keywords:** pain, tactile hypersensitivity, Chainmail Sensitivity test (CST), allodynia, spared nerve injury, axotomy, carrageenan, inflammation

## Abstract

Chronic pain occurs at epidemic levels throughout the population. Hypersensitivity to touch, is a cardinal symptom of chronic pain. Despite dedicated research for over a century, quantifying this hypersensitivity has remained impossible at scale. To address these issues, we developed the Chainmail Sensitivity Test (CST). Our results show that control mice spend significantly more time on the chainmail portion of the device than mice subject to neuropathy. Treatment with gabapentin abolishes this difference. CST-derived data correlate well with von Frey measurements and quantify hypersensitivity due to inflammation. Our study demonstrates the potential of the CST as a standardized tool for assessing mechanical hypersensitivity in mice with minimal operator input.

## Introduction

Pathological changes in the peripheral and central nervous system often lead to altered signaling, resulting in sensory symptoms not present in healthy individuals (Costigan et al., 2009). Painful symptoms include hypersensitivity to normally noxious stimuli (hyperalgesia) or previously innocuous stimuli (allodynia). Mechanical allodynia, pain elicited by touch, affects approximately 30% of patients with neuropathic pain (Jensen and Finnerup, 2014; Calvo et al., 2019), and most patients with chronic inflammation (Coutaux et al., 2005). Additionally, patients with nociplastic pain (chronic pain with no apparent stimuli) can present with either hyper- or hypo-tactile sensitivity (Kothari et al., 2015; Hilgenberg-Sydney et al., 2016). Together these changes in sensation can be extremely debilitating and represent a massive unmet clinical need (Costigan et al., 2009; Calvo et al., 2019). Increasingly, pain research in both patients and animals is becoming more categorized with respect to symptoms and signs, and more quantitative in its assessment (Calvo et al., 2019; Tappe-Theodor et al., 2019).

To date, the gold standard measuring technique of evoked tactile hypersensitivity in animals has been via calibrated von Frey monofilaments (Mills et al., 2012; Nirogi et al., 2012; Gonzalez-Cano et al., 2020), which are normally applied to the hind paw to determine withdrawal thresholds. Alternatively, an electronic version of von Frey filaments, the Dynamic Plantar Aesthesiometer, can be employed (Nirogi et al., 2012). Other approaches include assessing paw guarding (Cortright et al., 2008; Vierck and Yezierski, 2015) and dynamic or static weight bearing (Mogil et al., 2010; Piel et al., 2014; Suokas et al., 2014), using the CatWalk device (Vrinten and Hamers, 2003) or frustrated internal total reflection technology (FITR) (Zhang et al., 2022). Although with respect to body position multiple studies have not found a predictive role of gait in determining tactile sensitivity (Gabriel et al., 2009; Mogil et al., 2010; She et al., 2018), new approaches using video technology, for examining paw movement, represent an exciting new research avenue (Zhang et al., 2022; Bohic et al., 2023). Other pain-like sensitivity-measuring methods for rodents include self-administration of drugs (Bura et al., 2018) and place preference or aversion (King et al., 2009; Navratilov et al., 2013). Such procedures have significant utility because they measure the free will of the animal and eliminate the subjective analysis of the experimenter (Zhang et al., 2022). Beyond this, the experimenter can be removed, which calms the rodents considerably, improving data reliability. However, many of these methods require significant levels of learning, a major confound, both in terms of assay scalability and when chronic pain impairs cognitive function (Usdin and Dimitrov, 2016; Sieberg et al., 2018). Mostly for inflammation, natural rodent behaviors such as rearing (Matson et al., 2007; Bohic et al., 2023), burrowing (Andrews et al., 2012; Shepherd et al., 2018), hanging (Zhang et al., 2021) and facial expressions (Langford et al., 2010) have been used as markers (Tappe-Theodor et al., 2019). Interestingly, burrowing was the subject of the first multicenter clinical trial-like test of efficacy which demonstrated consistently positive results (Wodarski et al., 2016). Machine learning techniques applied to 3D video recording and site-specific electrophysiology has recently uncovered marked evoked ‘coping postures’ present in mice subject to carrageenan induced inflammation (Bohic et al., 2023) interestingly analgesics induce a new set of these spontaneous signatures which persist following resolution of evoked behaviors.

Despite multiple alternatives, von Frey filaments remain the predominant method of measuring laboratory-based tactile hypersensitivity. One reason for this is rodents are remarkably adept at compensating for paw injury and will avoid placing pressure on sensitive areas (Alexandrov et al., 2015). Therefore, a manually applied stimulus avoids this confound. Here, we introduce the Chainmail Sensitivity Test (CST). The CST studies behavior in freely moving mice, and results in an objective measurement of stimulus-induced tactile hypersensitivity that is simple to attain and replicate.

## Materials and methods

### Animal models

All animal procedures were approved by the Boston Children’s Hospital Animal Care and Use Committee, under animal protocol numbers 15-04-2928R and 16-01-3080R. All experiments were conducted blind to injury and treatment in a quiet room from 09:00 to 18:00. Male and female adult C57BL/6J mice (delivered 6–8 weeks for use at 8–10 weeks) were obtained from Jackson Labs (Maine, United States). Each behavioral test was performed on at least two independent cohorts; data were then merged.

#### Peripheral nerve injury

Mice were anesthetized with 3.5% isoflurane (vol/vol), anesthesia maintained at 2% isoflurane throughout the SNI surgery, which was performed at 7–9 weeks: The left tibial and common peroneal sciatic nerve branches were tightly ligated with a silk suture and transected distally, whereas the sural nerve was left intact (Decosterd and Woolf, 2000; Bourquin et al., 2006). For the sciatic nerve transection (axotomy), the left sciatic nerve was exposed at the mid-thigh level, ligated with silk, and sectioned distally. Sham-operated mice were subject to a similar surgery to the SNI mice. However, nerves were left untouched and the skin was closed with surgical clips (EZ Clips, Stoelting, Wood Dale, IL).

#### Drug administration (SNI)

Gabapentin (Sigma-Aldrich, Natick, MA) was dissolved in sterile PBS (Phosphate buffered saline) and administered intraperitoneally (IP) at 60 mg/kg. Vehicle controls were injected with an equal volume of PBS. Sham-operated controls were injected with an equal quantity of gabapentin or saline. Ibuprofen (Sigma-Aldrich) was IP injected at 30 mg/kg in PBS for associated trials. All mice were IP injected 30 min before being placed on the CST.

#### Carrageenan inflammation

Paw inflammation was induced with an intraplantar injection of carrageenan. Carrageenan solution (50 μL, 1% wt/vol in saline; Sigma-Aldrich) was freshly prepared on the day of the experiment, and injected into the plantar surface of the left hind paw using a microsyringe with a 30 gauge, ½ inch needle (Hamilton Company, Reno, NV) 4 hours before CST trials. Naïve saline controls were injected with an equal volume of saline at that time (Sigma-Aldrich). Ibuprofen was IP injected 30 min before CST trials. Ibuprofen-Carrageenan mice were injected to a final concentration of 30 mg/kg.

### Chainmail apparatus

**FIGURE 1:**
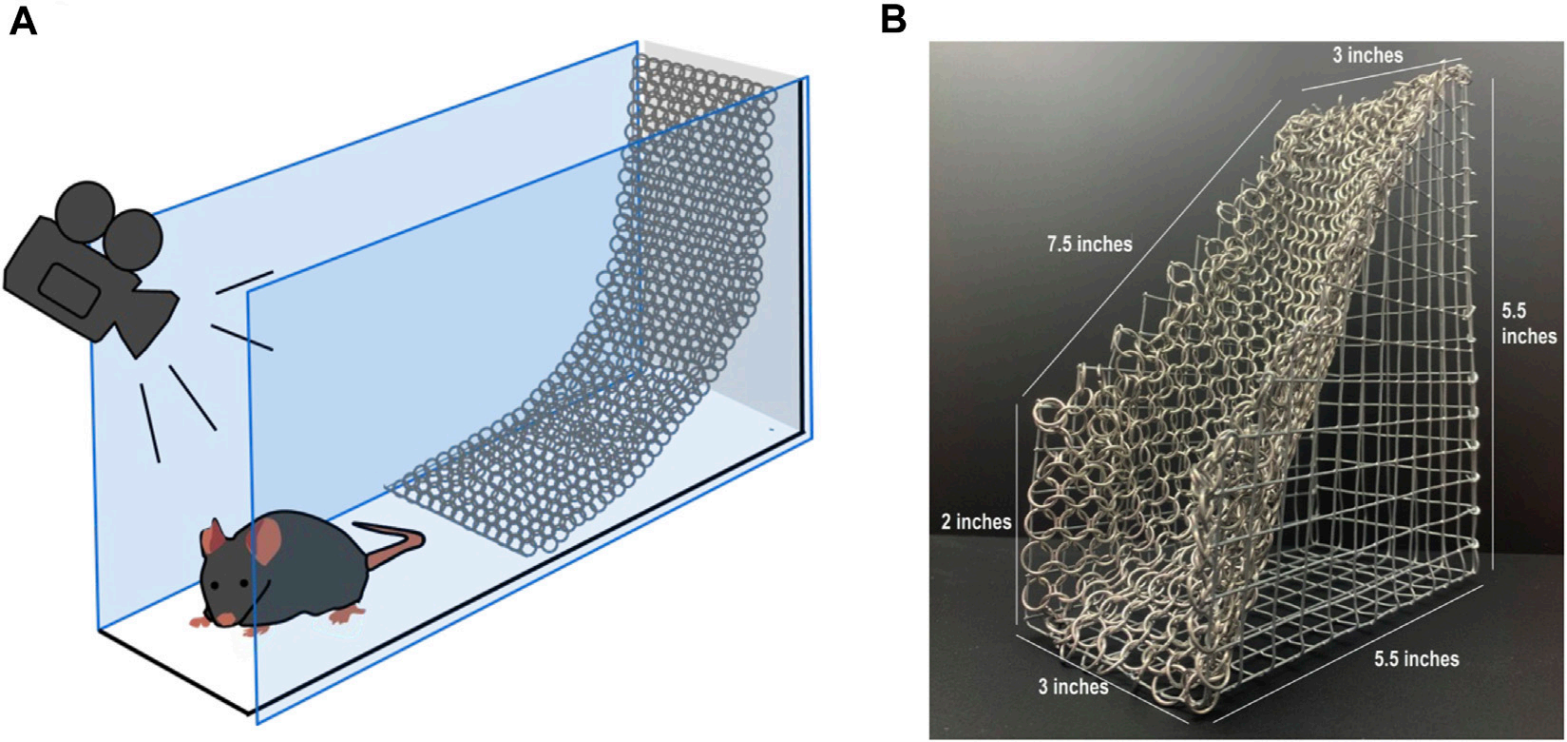
Chainmail sensitivity test (CST) device. **(A)** Schematic representation of the chainmail sensitivity test (CST) device, half of the chamber is occupied by an aluminum floor, while the other half is occupied by a chainmail surface hanging at a 45° incline from a galvanized steel wire support. The mouse can roam freely while its position is recorded by a camera above the apparatus. **(B)** Photograph of the chainmail section from the structure.

The CST apparatus consists of a galvanized steel wire support from which a chainmail hammock was hung at a 45° angle relative to the floor (Figure 1A). The device is positioned so that a mouse can freely climb up the chainmail or remain on an aluminum floor occupying equivalent space at the base of the CST structure. Mouse movement throughout the assay period was video recorded from above. The dimensions of the CST apparatus are provided (Figure 1B). Two adjacent CST devices are enclosed by Plexiglas walls, allowing the evaluation of two mice per trial (Supplementary Figure S1). The aluminum floor consists of the solid base of a Cold Hot Plate (Bioseb, Vitrolles, France). Each CST arena are separated by a matte black Plexiglas divider to prevent any visual or physical interactions between mice during testing.

## Measures of mechanical allodynia

### Chainmail sensitivity test

Mechanical allodynia was measured via the CST using the apparatus described above. Testing was conducted in a randomized manner, with animals from different groups being tested on the same day. Random number generation was used to allocate the order in which individual mice were tested. Animals were kept in a quiet room at room temperature at least 10 minutes before testing. Operators ensured no loud noises or rapid movements occurred while preparing mice. Cages were not changed or cleaned for at least 24 h leading up to testing. Unless required for drug administration, mice were not handled prior to use of the CST. At the start of each trial, mice were gently lifted and carefully lowered onto the aluminum floor side of the enclosed device, facing the chainmail. The investigator then started the video recording software and vacated the testing room. Animals were allowed to freely roam and explore the chamber for 30 min. Trials were recorded using an overhead EverFocus Ultra 720+ EQ(700) camera with a Tamron lens, mounted 1.524 m (5 feet) above the testing area. The chainmail was purchased from Amazon.com as a 8 by “6” rectangle cast iron cleaner.

### Video capture and processing

**FIGURE 2:**
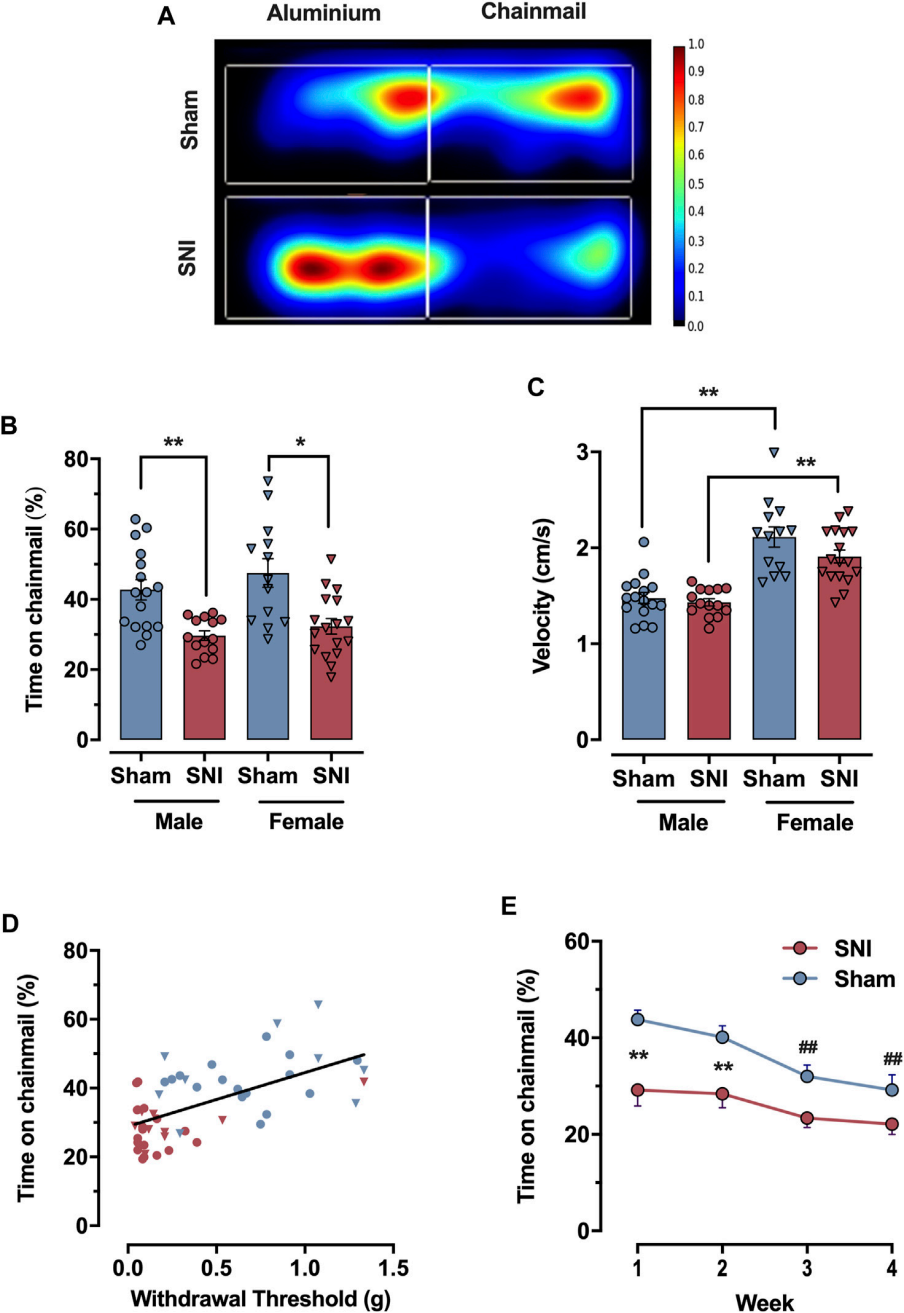
Testing neuropathic tactile sensitivity with the CST. **(A)** Heat map of average mouse movement on the CST (Sham (top), SNI (bottom), n = 5 per group merged). SNI mice prefer the aluminum surface, while sham-injured mice have no strong preference. **(B)** Percentage of time expended on the chainmail during the 30 min experiment for sham (blue) and animals subject to 7 days SNI (red), male and female animals (circles and inverted triangles respectively). **(C)** Mean velocity of the animals during the 30 min experiment, for sham (blue) and SNI (red), male and females (circles and inverted triangles respectively). **(D)** Linear correlation between 50% withdrawal threshold measured with von Frey filaments and percent time mice spend on the chainmail for SNI (red) and sham-injured (blue) male and female animals (circles and inverted triangles respectively). **(E)** Time-course of percentage of time expended in the chainmail after repeated exposures of 30 min once a week for sham (blue) and SNI (red) male animals. Each point represents the mean ± SEM of values obtained in 8–10 mice per group. Statistical significance for panel **(B)** and **(C)** determined by one way ANOVA followed by Bonferroni *post hoc* analysis; for **(E)** by 2-way ANOVA (mixed-model), followed by the Bonferroni *post hoc* analysis. Significant differences are denoted by * (*p* < 0.05), ** (*p* < 0.01) to indicate significant differences between groups at specific time points, while ## (*p* < 0.01) indicate significant differences over time within groups.

The video-recorded CST trials were tracked and saved on the Ethovision XT 11.5 software (Noldus, Leesburg, VA). Trials were recorded at 30 Hz for 30 min. Arenas and zones (for chainmail and aluminum) were delineated using the Ethovision software interface, as indicated in Supplementary Figure S1. Mice were tracked with center-point tracking (the “Differencing” option was selected with sensitivity set to 9). Summary statistics in 30-s time bins were automatically computed and exported as Microsoft Excel files. Position heat maps as seen in Figure 2A were generated using the same software package.

### Device cleanup and storage

Between trials, the aluminum floor, walls, and chainmail were cleaned with a damp disinfecting wipe (Peroxigard, Virox Technologies Inc., Canada), and were dried with paper towels. After completion of a trial session and at least daily, the chainmail apparatus and enclosing box were thoroughly washed with soap and water, and left to dry. We have found this cleaning protocol reduces experimental variance, especially in the control mice. We assume this variance is related to animal odor building up on the device.

### Data processing

The output files from Ethovision were processed by a custom-made program using Python 3.7. Numerical computations were performed using the NumPy package (Version 1.17.4) (van d et al., 2011). Features computed by the chainmail application for each trial include the total and cumulative amount of time spent on each surface, the average velocity of the animal, the total distance traveled, the number of independent entries onto each surface, and the average visit duration for each surface.

### Von frey filaments

Tactile sensitivity was measured at 7 days post-SNI in C57BL/6J mice using von Frey monofilaments (Touch-Test Sensory Evaluators; North Coast Medical, Inc., Gilroy, CA). Filaments used include 0.04, 0.07, 0.16, 0.4, 0.6, 1.0, and 2.0 g. Beginning with the 0.6 g filament, pressure was applied to the left hind paw three times from below, with 10 s intervals between each application. Depending on whether a response was elicited (brisk paw withdrawal and/or an escape attempt), a stronger or weaker filament was then used, following the up-down method as described in (Gonzalez-Cano et al., 2018).

### Carrageenan and paw volume

Paw volume measurements were made 3 h after initial intraplantar injection of either saline or carrageenan. Paw volume was measured using solution displacement quantified by a plethysmometer (Bioseb, Vitrolles, France). Paw volume measurements were repeated twice, with average displacement calculated.

### Multivariate analysis

PCA analysis was performed on the entire set of measurements described above using the Python SciPy library. A biplot was generated from this output to show separation of the data and the key contributors to such separation.

## Statistics

Prior to data analysis, any values found to be greater or smaller than two-times the standard deviation of the whole group mean were considered outliers and were removed (Wilcox and Keselman, 2003). Out of 412 unique values in the percentage of time on the chainmail, 14 were flagged as outliers according to the above criteria, or 3.4% of all values. After thorough analysis, we confirmed that our data fit a normal distribution. This assertion was validated using normality Kolmogorov-Smirnov tests. Given this normality, differences between the values were compared across experimental groups with one-way or two-way analysis of variance; with mixed-model, as appropriate, as indicated in the figure legends. Test were followed by the Bonferroni test, using the Prism 5 program (Graphpad Inc., La Jolla, CA, United States). The differences between means were considered statistically significant when *p* < 0.05.

## Results

### Quantification of neuropathic tactile sensitivity with the CST

The CST uses video tracking software to monitor a mouse’s preference between two equally-sized areas of an enclosed arena over a 30 min testing period. The first area is a plain metal floor which is slightly aversive for wild-type C57BL/6J mice at room temperature (22°C-25°C), as they prefer 33°C (Alexandre et al., 2017). The second area houses a chainmail hammock angled 45° to the floor, which mice may freely explore and climb (Figure 1A). Mice were tested 1 week after nerve injury, when tactile allodynia has fully developed (Cobos et al., 2018). Control mice unfamiliar with the CST are usually eager to climb on the chainmail and remain on it for 44.9% ± 2.4% (n = 29) of the testing period (male and female). In contrast, mice with a spared nerve injury (SNI) spend significantly less time on the apparatus, 31.1% ± 1.3% (n = 31); *p* < 0.0001, two tailed-test (male and female; Figures 2A, B). We propose that climbing on the free-moving chain links prompts mice to place weight on their injured paw, leading to hypersensitivity and consequently, avoidance. This contrasts with climbing on more rigid structures such as metal grids, where mice guard their injured extremity in an effort to prevent discomfort (Alexandrov et al., 2015).

The time spent on the chainmail showed significant differences between control (sham-injured) and SNI mice in both sexes. When results above are separated by sex, sham male mice spent 42.7% ± 0.4% (n = 16) of the time on chainmail, while the SNI group spent 29.7% ± 0.2% (n = 14) (*p* = 0.0038, one-way ANOVA, Bonferroni *post hoc*). In female mice, the sham group spent 47.5% ± 0.6% (n = 13) of the time on chainmail, and the SNI group spent 32.3% ± 0.4% (n = 17) (*p* = 0.018, one-way ANOVA, Bonferroni *post hoc*). However, no significant difference in time on the chainmail was observed between the sexes in sham mice or after injury (not significant, one-way ANOVA, Bonferroni *post hoc*, Figure 2B).

Despite this, female mice exhibited higher activity levels than male mice, moving faster both in the sham (1.48 ± 0.06 cm/s for males vs. 2.11 ± 0.11 cm/s for females; *p* < 0.0001, one-way ANOVA, Bonferroni *post hoc*) and SNI (1.43 ± 0.04 cm/s for males vs. 1.91 ± 0.07 cm/s for females; *p* < 0.0001, one-way ANOVA, Bonferroni *post hoc*, Figure 2C). Furthermore, female mice traveled greater distances throughout the assay period, regardless of injury type (Supplementary Figure S2A).

Given that the time spent on chainmail was similar between male and female groups, the overall increase in female mice’s activity was accompanied by shorter durations spent on the chainmail per visit (Supplementary Figure S2B).

### Time spent on the chainmail correlates with estimates of mechanical allodynia from von Frey filaments

To determine if time spent on the chainmail correlates with von Frey filament measures of tactile hypersensitivity, SNI and sham control mice were subjected to the CST and von Frey tests 1 week apart. Figure 2D shows that these two data sets are well correlated with *p* < 0.0001 and an R-squared value for the best fit slope of 0.364 (n = 48). As it is generally accepted that von Frey monofilament thresholds represent an accurate quantification of tactile sensitivity (Chaplan et al., 1994; Bourquin et al., 2006), we conclude that time spent on the chainmail represents a reliable inverse measure of stimulus-evoked tactile allodynia (Mogil et al., 2010).

### Repeated testing on the CST

To determine the behavior of male mice after repeated exposure to the CST apparatus, we placed sham and SNI-injured mice in the CST device weekly for 1 month. Sham-injured mice spend 43.8% ± 1.9% of their first exposure on the chainmail (n = 16), but this significantly decreases over repeated weeks, resulting in 29.2% ± 3.1% by Week 4, *p* < 0.01, two-way ANOVA (mixed-model, Bonferroni *post hoc*). SNI mice spend 29.2% ± 3.3% of time on the chainmail during their first exposure and 22.1% ± 2.1% of time by Week 4, a non-significant decrease (n = 16, Figure 2E). Between the SNI and sham groups, two-way ANOVA, mixed-model, with Bonferroni *post hoc* tests demonstrated a significant time difference in Week 1 (*p* = 0.0004) and Week 2 (*p* = 0.0033), but not in Week 3 (*p* > 0.05) or Week 4 (*p* > 0.05).

### Effect of gabapentin on time spent on chainmail following SNI

**FIGURE 3:**
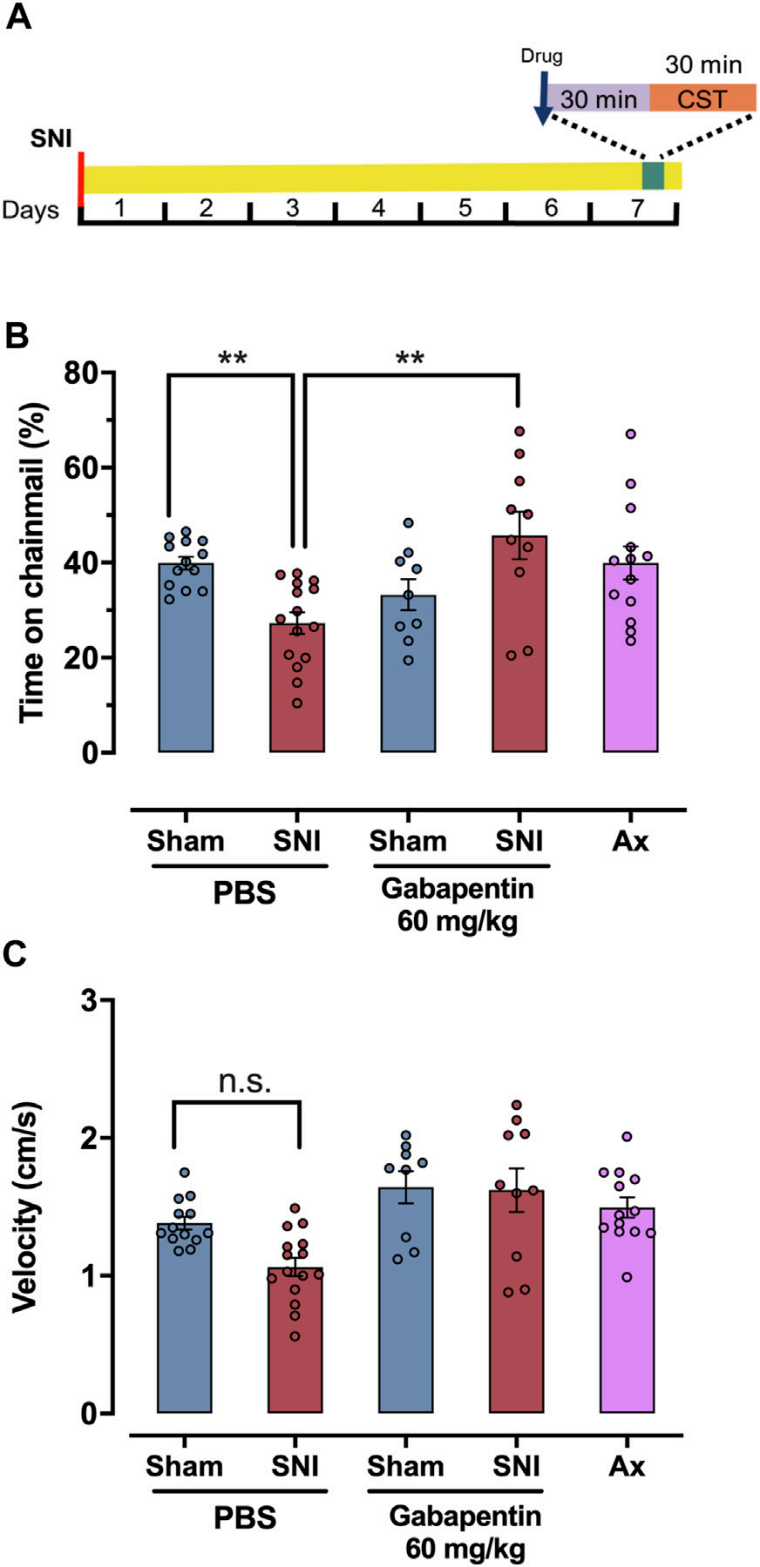
Gabapentin reverses SNI mice reticence to climb on the chainmail. **(A)** Schematic representation of treatment protocol for SNI, sham or axotomy animals treated with drugs or solvent. The spared nerve injury (SNI) or axotomy was performed 7 days prior to the experiment, and the drug or solvent was administered 30 min before the test. **(B)** Response in a chainmail sensitivity test after treatment with PBS, gabapentin 60 mg/kg in SNI, sham and axotomy animals. **(C)** Average velocity (cm/s) in a chainmail sensitivity test after treatment with PBS or gabapentin 60 mg/kg in SNI, sham and axotomy mice. For **(B)** and **(C)** individual values are represented as points. One-way ANOVA was used for statistical analysis, and significant differences are indicated by * (*p* < 0.05), ** (*p* < 0.01).

Gabapentin is a first line anti-neuropathic pain agent in humans (Gilron et al., 2015; Finnerup et al., 2018) and multiple studies have shown it is effective in relieving chronic neuropathic hypersensitivity in rodents (von Hehn et al., 2012). Here we tested if gabapentin could reduce the reticence of male mice to explore the chainmail side of the CST device 7 days after SNI. Gabapentin (60 mg/kg) dissolved in saline was IP injected 30 min before testing, while vehicle control mice were injected with saline (Figure 3A). The standard dose range of gabapentin used in von Frey-based rodent assays of neuropathic allodynia is 50–100 mg/kg (Field et al., 2000; Patel et al., 2001; Joshi et al., 2006). SNI-injured mice treated with gabapentin (60 mg/kg) (n = 10), spent 45.7% ± 5.0% of assay time versus 33.26% ± 3.2% for gabapentin-treated sham controls (n = 9), not significant, one-way ANOVA, Bonferroni *post hoc*, Figure 3B).

### Effect of axotomy on time spent on the chainmail

To control for the possibility that SNI mice do not climb on the chainmail portion of the CST apparatus because of a lack of motor control brought on by nerve injury, we tested male mice subject to a full sciatic nerve axotomy on the CST. Fully axotomized mice lack any evoked sensation within the denervated hind paw, save the saphenous territory (Hsieh et al., 2000). At 7 days post injury no directly injured sensory axons remain in the lower paw. In addition, motor control is profoundly affected, with little or no movement possible in the lower leg. The saphenous does not contain any motor fibers and remains uninjured. Conversely, mice subject to SNI retain some innervation from the spared sural nerve which, with respect to the sensory system, is responsible for the tactile allodynia present, and within the motor system allows for a minimal level of movement. Mice subject to complete axotomy of the sciatic nerve (n = 13) explored the chainmail 39.9% ± 3.5% of the assay time, compared to 44.9% ± 1.9% for sham control mice (n = 13) and 27.3% ± 2.3% for SNI mice (n = 15). There was no significant difference between sham and axotomy groups, though there was a significant difference between axotomy and SNI group, (*p* = 0.01, one-way ANOVA, Bonferroni *post hoc*, Figure 3B).

### SNI does not alter level of activity

To explore the possibility that the SNI animals do not move due to pain or anhedonia, we quantified the mean velocity by these mice and compared it to the sham-injured animals (Figure 3C). PBS-injected mice subject to SNI had a velocity of 1.06 ± 0.07 cm/s (n = 15), while sham mice 1.38 ± 0.05 cm/s (n = 13), not significant (one-way ANOVA, Bonferroni pos hoc). At 60 mg/kg of gabapentin, sham mice (n = 9), and SNI groups (n = 10), had a slightly greater level of activity (1.64 ± 0.12 cm/s and 1.62 ± 0.16 cm/s, respectively) than the saline injected controls, though neither change was significant (one-way ANOVA, Bonferroni *post hoc*). Mice subject to sciatic nerve axotomy had a velocity of 1.49 ± 0.07 cm/s (n = 13), which was also not significantly different from the SNI and sham-injured mice (one-way ANOVA followed by Bonferroni *post hoc* test). This indicates that the SNI, axotomy and sham-injured males are equally active. Additionally, distance (Supplementary Figure S3A), average time per chainmail visit (Supplementary Figure S3B) and the number of visits to the chainmail (Supplementary Figure S3C) did not differ among the groups above.

### Principal component analysis

**FIGURE 4:**
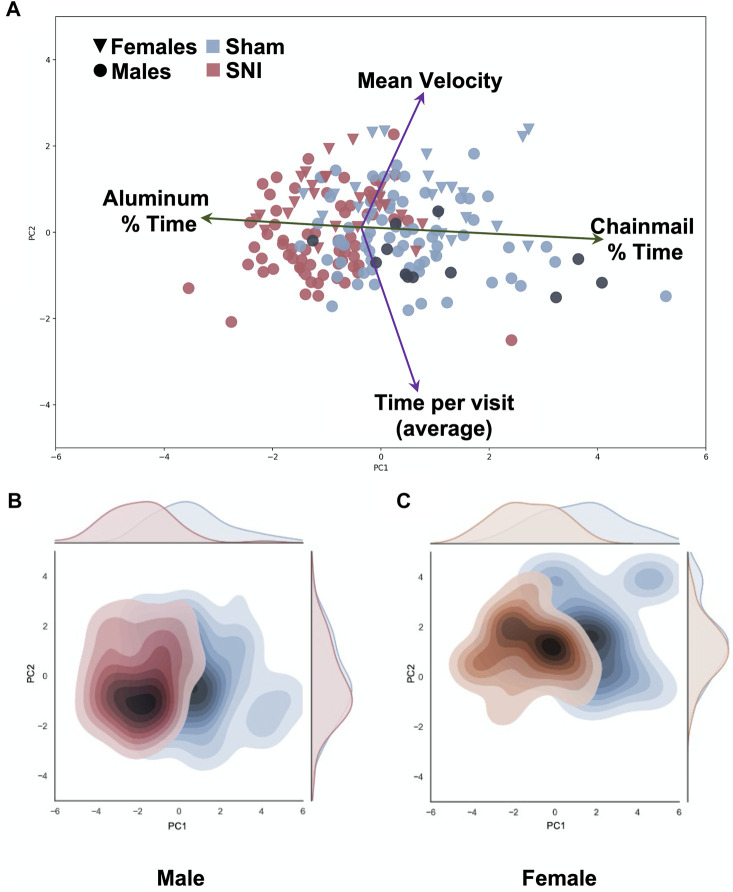
Multivariate Principal Component analysis of SNI and sham data. Variables include chainmail % time, aluminum % time, mean velocity, average time per visit (chainmail or Aluminum). Principal Component 1 (PC1) on the *x*-axis demonstrate SNI mice (red) spend more % time on the aluminum surface compared to sham control mice (blue) (Explained variance ratio: 47%). Principal Component 2 (PC2) on the *y*-axis demonstrates variation in average velocity between female (triangle) and male (circle) groups (explained variance ratio: 23.3%). Gabapentin-treated SNI male mice, noted by black circles, demonstrate that these points mainly distribute with the sham-injured points. **(A)**. Heat map of the distribution of male mice demonstrate no difference in velocity (PC2) between sham and SNI groups **(B)**. Heat map of the distribution of female sham and SNI mice demonstrates a separation toward higher mean velocity along PC2 **(C)**.

Multivariate analysis demonstrates that time spent on the chainmail *versus* time on the aluminum surface effectively separates SNI from sham injured values (Figure 4A; Supplementary Figure S4A). Interestingly male and female mice separate in a manner consistent with mean velocity and time per visit (Figures 4B, C) as described above. Highlighting SNI mice treated with 60 mg/kg gabapentin on this plot shows that these mice, in the main, separate with sham injured animals (Figure 4A, black points) as was the case with naive mice (Supplementary Figure S4B). The variance in the plotted data is primarily accounted for by the first (PC1) and second (PC2) principal components, which contribute 50% and 24% respectively (Supplementary Figure S4C).

### Time spent on the chainmail in inflammatory pain

**FIGURE 5:**
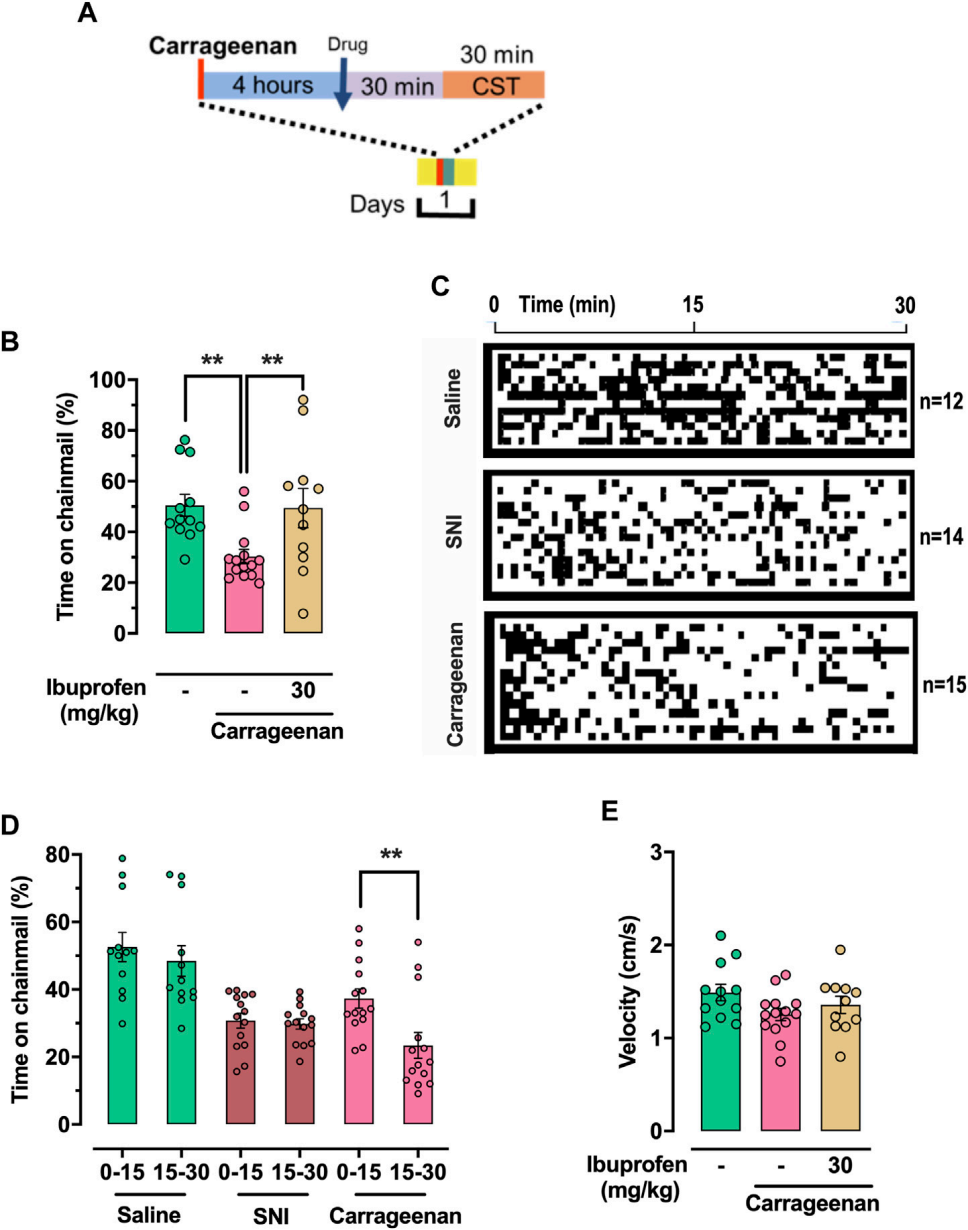
CST quantification of tactile hypersensitivity in carrageenan-induced inflammation. **(A)** Schematic representation of treatment protocol for carrageenan-inflamed mice treated with drugs or solvent. Carrageenan-treated mice were injected in the left hind paw with 50 µL carrageenan, 1% wt/vol in saline, 4 h before being placed on the CST. Drug were dissolved in PBS and administered intraperitoneally 30 min before being tested on the CST. **(B)** Percentage of time expended in the chainmail during the 30 min experiment. **(C)** Mouse behavior represented as a function of time. Black represents time on chainmail; white represents time on aluminum. The behavior of individual animals is represented as vertically stacked horizontal bars. **(D)** Percentage of time expended in the chainmail during the 30 min experiment, separated in 15 min segments. **(E)** Mean velocity of the animals during the 30 min experiment. For **(B)**, **(D)** and **(E)** individual values are represented as points. One-way ANOVA **(B)** or 2-way ANOVA (mixed-model) **(D)** were used for statistical analysis, and significant differences are indicated by ** (*p* < 0.01).

Here we examined quantification of carrageenan-induced hypersensitivity in male mice (Winter et al., 1962; Morris, 2003). Carrageenan solution (50μL, 1% wt/vol in saline) was injected into the plantar surface of the left hind paw 4 hours prior to the CST, while vehicle control mice were injected with saline. Mice treated with ibuprofen at 30 mg/kg were injected 30 min before the assay (Figure 5A). Carrageenan-treated mice (n = 15) spent an average of 30.4% ± 2.6% of the assay time on the chainmail *versus* 50.5% ± 4.3% for naïve-PBS (saline-injected) mice (n = 12), (*p* = 0.006, one-way ANOVA, Bonferroni *post hoc*, Figure 5B). In addition, carrageenan-inflamed mice treated with ibuprofen (n = 11) spent 49.4% ± 7.7% of the assay time on the chainmail, which is not significantly different to naïve-saline controls (one-way ANOVA, Bonferroni *post hoc*). These data provide evidence of the relationship between the time spent on the chainmail and tactile hypersensitivity due to peripheral inflammation. In order to demonstrate peripheral edema due to carrageenan-induced inflammation, we measured paw volume 3 h after carrageenan injection. Carrageenan mice (n = 15) measured 0.188 ± 0.006 mL, which was significantly higher than the 0.103 ± 0.007 mL measured in naïve-saline mice (n = 14), (*p* < 0.0001, two-tailed *t*-test, Supplementary Figure S5A).

### Differences in chainmail climbing behavior between SNI and carrageenan mice

By examining the time spent on the chainmail during a trial (Figure 5C), we identified a difference in the behavior of the SNI mice relative to carrageenan-inflamed male animals. This was examined as time spent on the chainmail in the first 15 min of the assay period relative to the final 15 min. In the carrageenan mice (n = 15), there was a significant difference in the time spent on the chainmail between these two periods, with mice spending more time on the chainmail in the first 15 min window (Carrageenan 0–15 min 37.7% ± 2.7%; carrageenan 15–30 min 23.0% ± 3.6%; (*p* > 0.0001, 2-way ANOVA mixed-model, Bonferroni *post hoc*). This is in contrast to the SNI and sham mice, where no such difference between these two periods exist (Figure 5D). We assume this difference in CST exploration in acutely inflamed mice is due to sensitization of the peripheral inflamed tissue of the paw, which results in the mice becoming less willing to climb on the chainmail as time progresses.

Neither velocity (Figure 5E), distance traveled (Supplementary Figure S5B), nor time per chainmail visit (Supplementary Figure S5C) were significantly different after carrageenan injection or subsequent ibuprofen treatment. Carrageenan-treated mice (n = 15) visited the chainmail 53.1 ± 3.8 times during the 30-min trials, significantly less than 74.3 ± 4.7 visits for saline-injected controls (n = 12), (*p* = 0.049, one-way ANOVA followed Bonferroni *post hoc*, Supplementary Figure S5C).

## Discussion

Little has changed for over one hundred years in the quantification of tactile sensitivity in laboratory animals. In 1896, Maximilian von Frey introduced graded filaments to test mechanical hypersensitivity. Although heavily used and considered the gold standard for measuring tactile sensitivity in both experimental animals and patients, von Frey monofilaments have multiple disadvantages (Bove, 2006). First, the von Frey method requires a highly trained operator and therefore can be expensive to perform both in terms of time and outlay. Additionally, methods that rely on direct human quantification will always be subjective and potentially error prone.

Another problem with von Frey testing is differences in scoring techniques between investigators leading to results that are equally valid but cannot be cross-compared. For instance, the 50% threshold method is a popular means of analyzing von Frey thresholds (Chaplan et al., 1994). However, different definitions of a positive response are used between labs, such as 5 responses per 10 stimulations *versus* 1 of 3 stimulations. The time between each filament stimulation can also vary significantly between labs from 2 to 30 s, potentially altering the level of sensitization the animal experiences from the test itself (Xie et al., 2015; Mangione et al., 2016; Cobos et al., 2018). In addition, not all von Frey fibers are made to the same specification, hampering cross-comparison of data between research centers. Furthermore, von Frey filaments should be, but frequently are not, calibrated regularly to prevent error. Other issues with von Frey testing include that the rodents are usually placed on a metal grid to allow access to the paws, which is uncomfortable and can lead to sensitization (Pitcher et al., 1999). Mice often require relatively long periods of habituation on this grid, further amplifying this potential sensitization. Finally, the presence and even the gender of the experimenter in the room can influence animal behavior (Sorge et al., 2014), and therefore removing the operator altogether during the testing period would be optimal. To this end adding the CST device to the home cage would be a potential next step in normalizing testing conditions further. Mouse sensitization and differences in von Frey protocols have together resulted in a problem of translating von Frey results in both research laboratory settings (Percie du Se et al., 2014) and the pharmaceutical arena (Whiteside et al., 2013; Barrett, 2015). Despite these issues, the von Frey method when performed carefully is a reliable, if cumbersome, means of quantifying tactile sensitivity (Bradman et al., 2015).

We set out to create a novel test of tactile allodynia in mice, one that is easy to perform and replicate. By defining the differential amount of time spent on either a plain metal floor or the chainmail structure by video tracking, we are able to automate and accelerate data collection whilst removing potential bias. Some have postulated that spontaneous pain would be the best parameter to test in mice to most accurately mirror neuropathic patient symptoms (Percie du Se et al., 2014). With no means of directly communicating with the experimental animals, accurately determining such sensory information is complex (Mogil et al., 2010). Therefore, stimulus-evoked hypersensitivity is a useful proxy for the general discomfort of chronic pain. In addition, stimulus-evoked mechanical pain is a major disabling symptom suffered by approximately 30% of neuropathic patients (Percie du Se et al., 2014) and by most chronic inflammatory patients (Coutaux et al., 2005). Studying mouse behavior using an experimental paradigm which allows a choice of response will always be preferable to one which confines an animal and provides limited response options. Indeed, von Frey filaments contain a spinally mediated reflex component. The chainmail test requires active decision making to avoid noxious stimulation, involving cerebral processing, these mechanisms represent a more central focus on brain processes that add to translation between this assay and patients. Methods that encompass this parameter include place preference/aversion (Cunningham et al., 2006; Zhang et al., 2022). These approaches have been used successfully in chronic pain studies including neuropathic sensitivity assays (King et al., 2009). One limitation of these methods, however, is the requirement of a learning aspect, which adds to the tests complexity and may be a serious confound, especially in chronic pain which can significantly alter cognition (Usdin and Dimitrov, 2016). The CST device allows the mice to freely choose where to wander within the apparatus, however, the tactile response it measures is evoked, creating an immediate response.

Results demonstrating gabapentin-mediated analgesia of SNI-induced hypersensitivity or ibuprofen-mediated analgesia in carrageenan-induced inflammation suggest that analgesic compounds can be accurately studied using the CST. As we demonstrate a significant correlation with von Frey stimulation in neuropathic (SNI) and control mice, we assume that we are indeed measuring touch-evoked hypersensitivity in the animals rather than another central pain phenomenon such as sickness syndrome (Saper et al., 2012). Sickness syndrome is analgesic responsive, but is distinct from injury induced-allodynia. Therefore, it is important to accurately define which “pain-like” response is being assayed to avoid false positive data and confusion in attempting to delineate specific mechanisms (Calvo et al., 2019). Indeed, other authors have noted strong “pain-like” effects that do not correlate with hind limb sensitivity as measured by von Frey filaments, in both the burrowing assay (Andrews et al., 2012) and the CatWALK device (Mogil et al., 2010). It is also unclear what specific pain modality place preference/aversion tests are measuring (Calvo et al., 2019). It should be noted that the implementation of machine learning tools on the videos obtained in von Frey experiments (Jones et al., 2020) or other technologies as the use of frustrated total internal reflection technology (Zhang et al., 2022) or 3D imaging (Bohic et al., 2023) greatly enhances their potential. These machine learning tools can also be applied to CST videos to increase the information obtained from them.

One limitation of the CST approach is that this method cannot be repeated multiple times with the same mice without a reduced level of exploration on the chainmail side of the device (Figure 2E). It is difficult to solve this problem in methods that assay free will in experimental animals (Matson et al., 2007). SNI mice, however, are sensitive to the chainmail from the start, and remain this way throughout the experiment. Even after multiple sessions on the CST device, mice subject to SNI still investigate the chainmail approximately 25% of the testing period. This suggests that this number represents the lower end of activity on the chainmail portion of the device using this model. One possible reason for the continued interest in exploring the chainmail for 25% of the time is that the animal’s strong desire to explore and determine an escape route outweighs even learnt aversion to climbing on the chainmail. This hypothesis is supported by the in-depth analysis of SNI climbing behavior shown in Figure 5C, which demonstrates very short climbing bouts on the chainmail. Continuing reticence to climb the chainmail in injured mice may open this approach up to large scale drug screening programs by using multiple devices and mice.

We introduce a novel method for measuring experimental mechanical allodynia, which leverages free movement in mice as its readout. The CST device is sensitive and can differentiate between mice experiencing mechanical hypersensitivity using several distinct pain models. This method provides distinct advantages over von Frey testing, including the removal of the investigator, variability between researchers, intensive investigator training, and unintentional animal sensitization. Together, these advantages can increase data reliability which in turn can lead to more accurate conclusions of analgesic treatment trials and better drug screens. Such improvements should result in better outcomes for chronic pain patients who are experiencing a dearth of effective analgesics.

## Data Availability

The raw data supporting the conclusion of this article will be made available by the authors, without undue reservation.
