# Supplementary figures and images for: Quantification of stimulus-evoked tactile allodynia in free moving mice by the chainmail sensitivity test
Source: Front Pharmacol. 2024 Feb 23;15:1352464. doi: 10.3389/fphar.2024.1352464 (PMC10920263; doi:10.3389/fphar.2024.1352464)

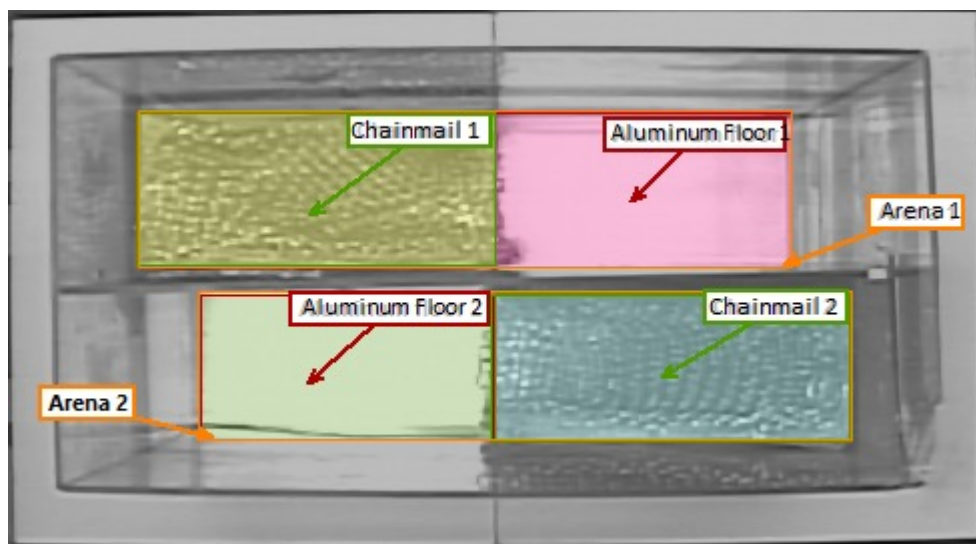

**A**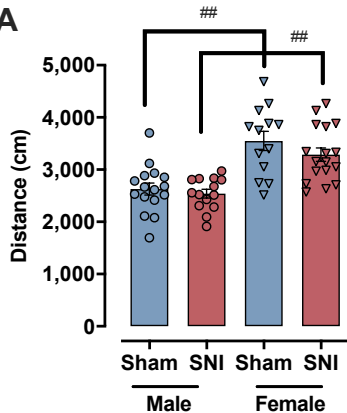**B**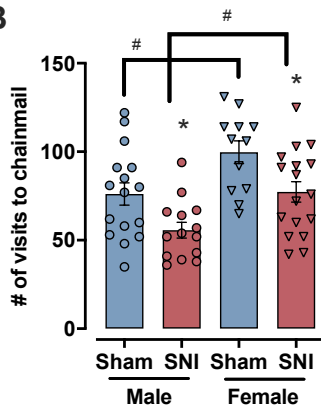**C**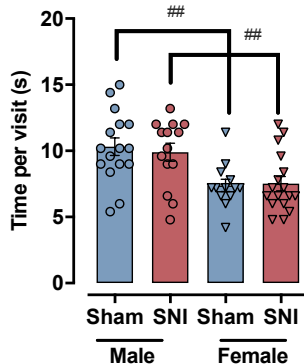

A

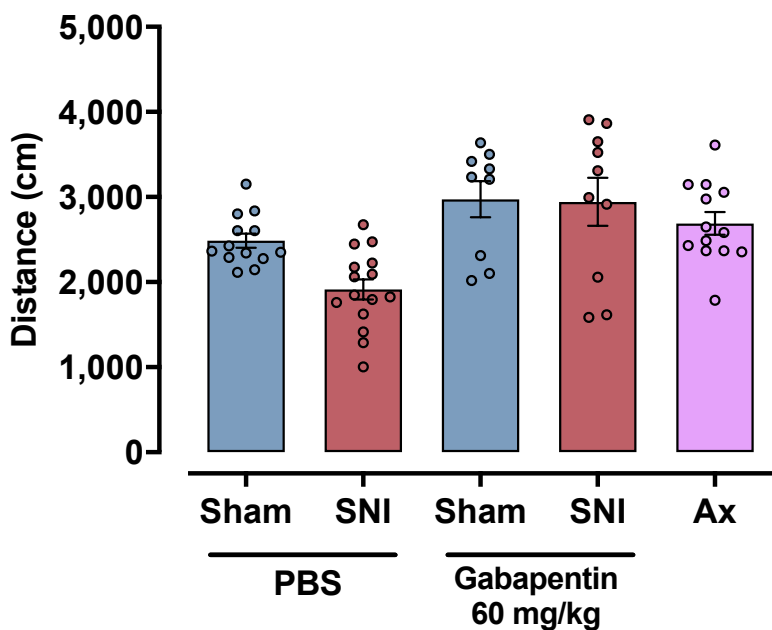

B

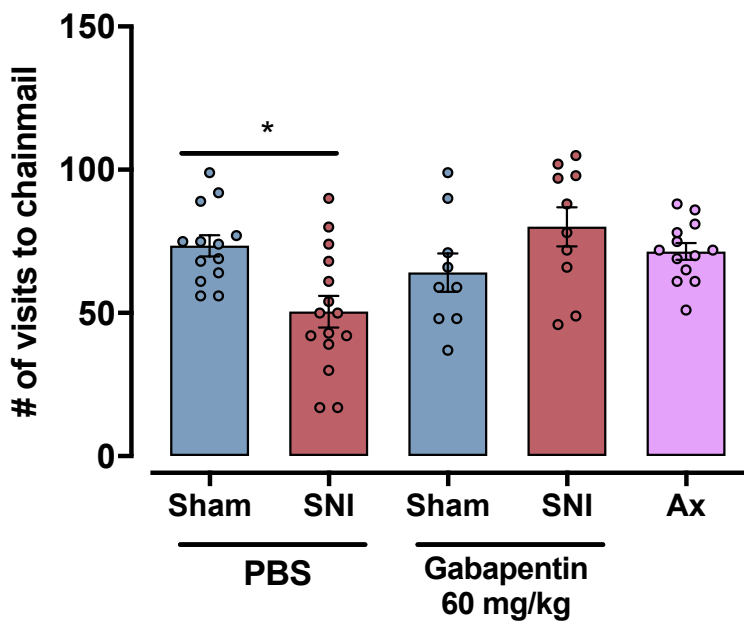

C

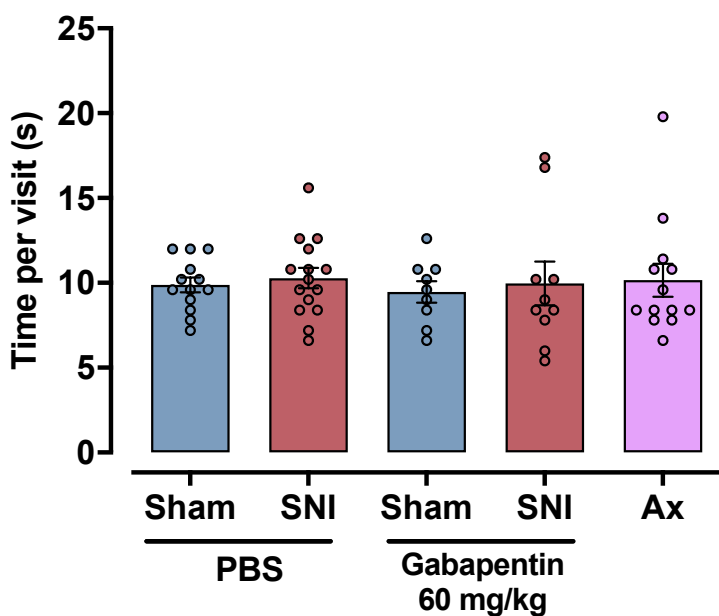

**A**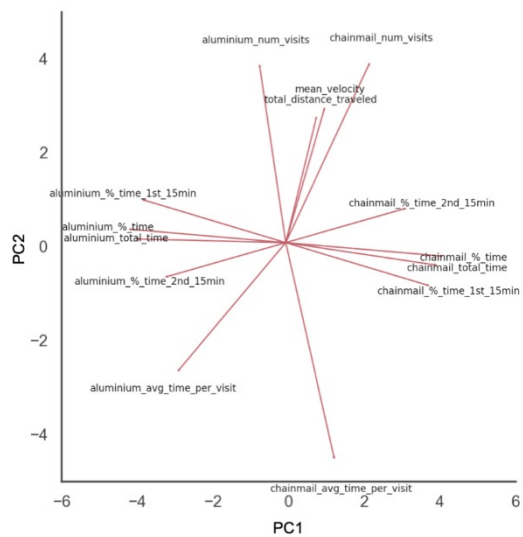**B**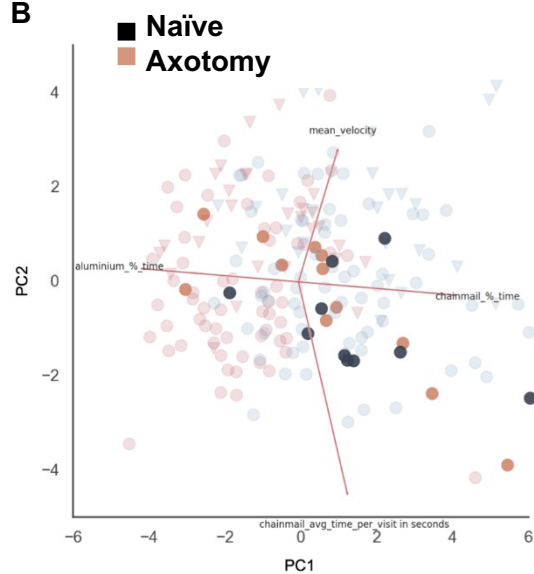**C**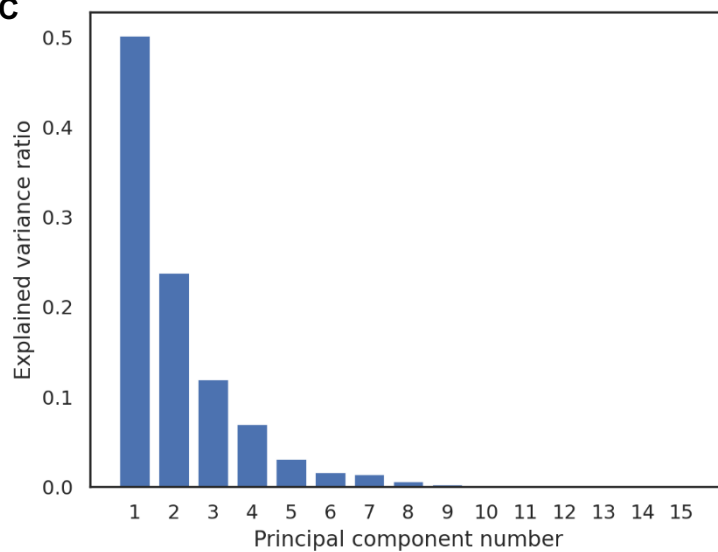

A

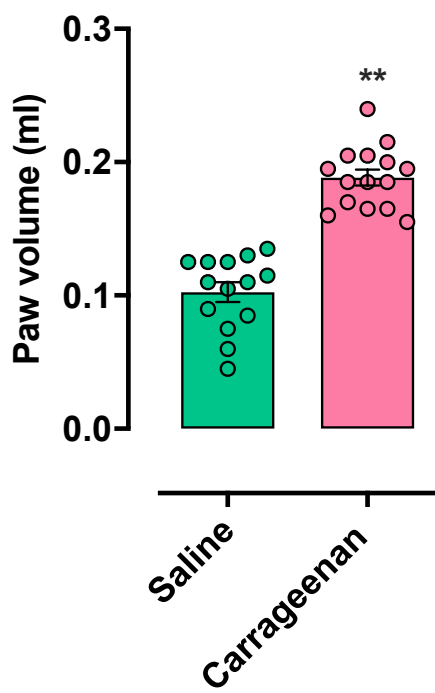

B

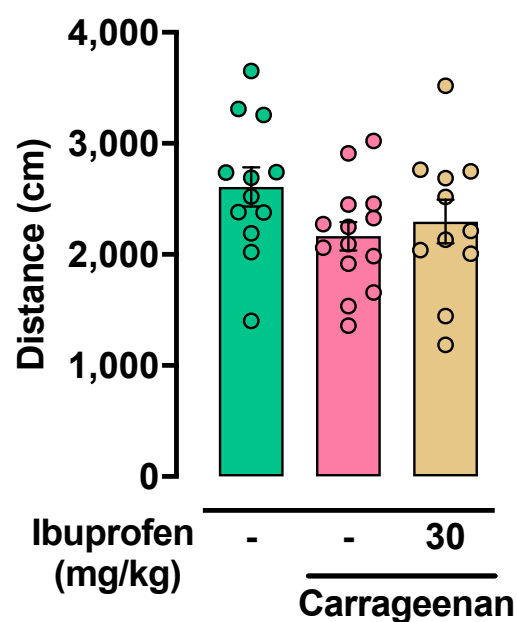

C

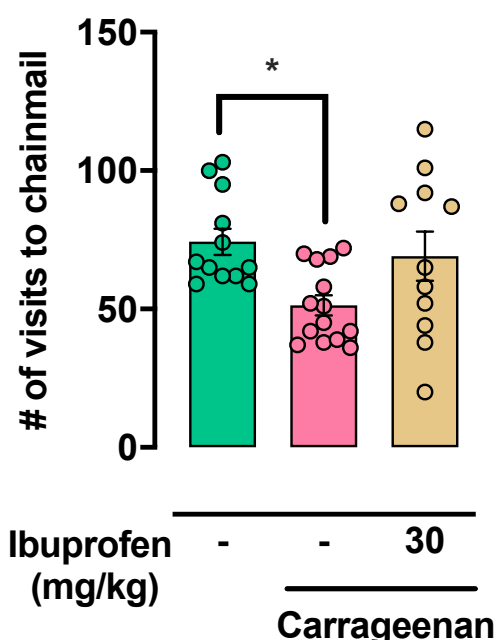

D

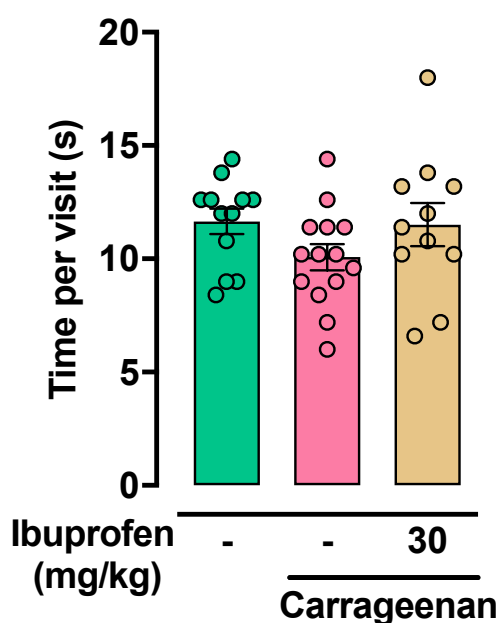

Supplement: Supplementary file 1 [file DataSheet1.PDF]
